# The Role of Large-Scale Data Infrastructure in Developing Next-Generation Deep Brain Stimulation Therapies

**DOI:** 10.3389/fnhum.2021.717401

**Published:** 2021-09-06

**Authors:** Witney Chen, Lowry Kirkby, Miro Kotzev, Patrick Song, Ro’ee Gilron, Brian Pepin

**Affiliations:** ^1^Rune Labs, San Francisco, CA, United States; ^2^Department of Neurological Surgery, University of California, San Francisco, San Francisco, CA, United States

**Keywords:** big data, precision medicine, data infrastructure, neuromodulation, deep brain stimulation

## Abstract

Advances in neuromodulation technologies hold the promise of treating a patient’s unique brain network pathology using personalized stimulation patterns. In service of these goals, neuromodulation clinical trials using sensing-enabled devices are routinely generating large multi-modal datasets. However, with the expansion of data acquisition also comes an increasing difficulty to store, manage, and analyze the associated datasets, which integrate complex neural and wearable time-series data with dynamic assessments of patients’ symptomatic state. Here, we discuss a scalable cloud-based data platform that enables ingestion, aggregation, storage, query, and analysis of multi-modal neurotechnology datasets. This large-scale data infrastructure will accelerate translational neuromodulation research and enable the development and delivery of next-generation deep brain stimulation therapies.

## Introduction

Precision medicine has changed the face of modern healthcare. Historically, treatments have been developed assuming a one-size-fits-all approach. Now, thanks to advances in multi-modal data collection and analysis, we understand that many diseases are heterogeneous and require personalized treatment strategies. Precision oncology has been at the forefront of this revolution. High-throughput technologies generating large, multi-omics datasets have spurred data-driven approaches to inform risk prediction, disease detection, diagnosis, phenotyping, and identification of new therapeutic targets. Data aggregation platforms emerged as critical tools for structuring and sharing these complex data, driving data utility for both the researchers and clinicians who are developing next-generation, precision therapies (de Anda-Jáuregui and Hernández-Lemus, 2020).

In neurological and psychiatric disorders such as Parkinson’s disease (PD), epilepsy, major depressive disorder, and obsessive compulsive disorder, data-driven approaches for disease classification and treatment have yet to gain widespread integration into clinical decision making. Each diagnosis remains a heterogeneous mixture of phenotypes, with limited options for personalized therapies. However, several studies have demonstrated the value of characterizing patient-specific disease pathophysiology. For example, studies utilizing neuroimaging and neurophysiology have identified putative subgroups of depression, which may be valuable in predicting responsiveness to therapy (Riva-Posse et al., 2014; Drysdale et al., 2017; Williams, 2017; Wu et al., 2020). These initial studies illustrated the utility of a single cross-sectional snapshot of neural circuitry, but acute assessments fail to capture the full complexity of these time-varying disorders.

Transitioning research outside of the acute clinical setting and into longitudinal real-world environments requires tools that can track both neural activity and patient clinical state over the span of years. Implantable neural devices have enabled chronic field potential recordings in brain circuits, with the potential to continuously stream data over the lifetime of the device (Stanslaski et al., 2018; Gilron et al., 2021; Goyal et al., 2021). However, data labeling and contextualization are important for maximizing the utility of these electrophysiological recordings. Thus, simultaneous monitoring of patient state is critical. Digital technologies such as wearable sensors, as well as clinician assessments and patient self-report, provide both objective and subjective measures of patient state (Bot et al., 2016; Pathak et al., 2021; Powers et al., 2021).

The resulting large-size, multi-modal datasets require significant data infrastructure that supports scalable data ingestion, time-syncing, storage, query, and analysis. These systems are complex from a technical, reliability, and compliance standpoint and are beyond the capacity of most individual research groups. Rune Labs has developed a data platform that is uniquely tailored to the needs of the neuromodulation community. We present this as an example of the type of infrastructure that can be used to develop and deliver data-intensive neuromodulation therapies. We discuss advantages of using a common infrastructure, which include ease of data sharing and replication of results, both within and across teams.

## Unmet Needs in Precision Neuromodulation

Longitudinal neural physiology combined with objective monitoring of clinical state is particularly important for disorders that are time-varying. For example, in PD, patients fluctuate between periods of adequate and inadequate symptom control, as dopaminergic medications wear in-and-out (Kalia and Lang, 2015). Conventional deep brain stimulation (DBS) delivers continuous stimulation to the basal ganglia nuclei, regardless of a patient’s clinical state. This can lead to both under-stimulation, resulting in inadequately controlled symptoms, or over-stimulation, resulting in unwanted side effects such as dyskinesia (Beudel and Brown, 2016). Adaptive DBS (aDBS) aims to address these shortcomings by using biomarkers to track disease fluctuations and update therapy delivery in real-time. Acute tests of aDBS have demonstrated the feasibility of incorporating a feedback signal into stimulation titration, and studies have matched the clinical efficacy of continuous DBS (Little and Brown, 2020). However, these studies have been limited to short clinical visits, and prolonged tests of aDBS have not yet been shown to be more efficacious than standard DBS (Gilron et al., 2021).

Formulating aDBS paradigms that translate outside of the clinic is first dependent on identifying biomarkers that track clinical state. In PD, several biomarker candidates have emerged that track the hypo- and hyperkinetic states, though they have yet to be validated in chronic settings (Little and Brown, 2020). Similarly in depression, candidate neurophysiological biomarkers have been identified solely in acute settings (Kirkby et al., 2018; Sani et al., 2018; de Aguiar Neto and Rosa, 2019). Accordingly, researchers are adapting their data collection and analysis protocols for longitudinal, at-home recording. These recordings capture the full spectrum of clinical variability and naturalistic human behaviors that cannot be assessed in clinic. Importantly, generating insights from these rich neural time series requires precise integration with other at-home monitoring data, including wearable sensor time series and single time-point reports of patient state. Thus, drawing the links between patient state and neural physiology is dependent on being able to access precisely synchronized data from several data streams.

Furthermore, testing aDBS over long time courses in patients’ homes requires efficient data transfer and availability. Both researchers and clinicians need easy access to recorded data such that they can assess algorithm performance and iterate on tests. This involves transferring data from the patient’s implanted device to external computers and/or cloud based storage without requiring frequent clinic visits. This transfer must be both efficient and secure. Data from raw device files must then be parsed and represented in an accessible manner to both researchers and clinicians, and these analysis pipelines must be integrated into their normal workflows.

Finally, optimizing aDBS can benefit from data-driven approaches to streamline the programming process. aDBS optimization is currently constrained by a large parameter search space, in part due to increasingly sophisticated electrode designs and stimulating device capabilities. Furthermore, individual variability in electrode locations and neuroanatomy is not taken into account. Data-driven modeling frameworks can be explored to narrow aDBS tuning parameters and guide clinical programming. These modeling approaches are contingent on the ability to query and aggregate large datasets and conduct computationally intensive analyses.

Thus far, lags in technological development have delayed large-scale testing of aDBS in chronic settings. Capabilities of earlier generation implantable neural devices initially limited the ability to do biomarker discovery. As new devices pushed brain sensing into patients’ homes, there became an additional need for tools that chronically evaluate patient state. Developing patient- and researcher-facing applications, in addition to data infrastructure that integrates these large data sets, requires software engineering resources and time. Thus unlocking the full utility of these rich datasets requires a scalable and efficient data platform.

## A Data Infrastructure Solution

Rune Labs has developed a scalable, HIPAA-compliant, cloud-based data platform designed for (1) time-synchronization and aggregation of multi-modal datasets (2) real-time data access, and (3) data analysis at the scale of multiple terabytes, directly in the cloud. The platform is optimized for datasets generated in neuromodulation research, such as longitudinal time series data from a variety of devices, including but not limited to neural implants, wearable sensors, and patient-facing phone-based applications.

### System Architecture

**FIGURE 1:**
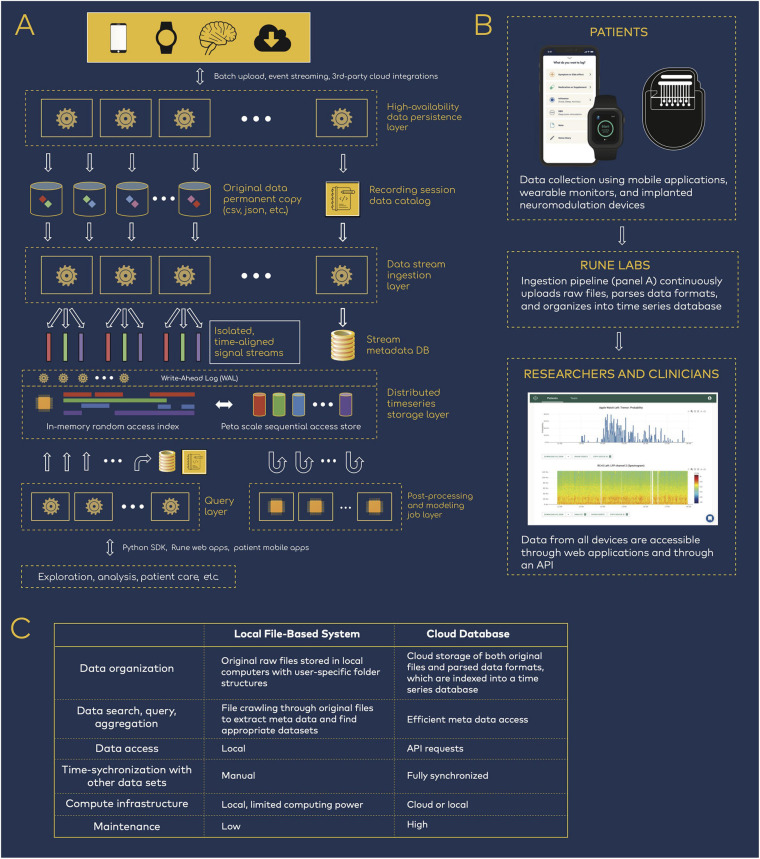
Data infrastructure for neuromodulation research. **(A)** Rune system architecture. Patient data spanning neural physiology, wearable physiology, and mobile applications step through several processing layers that parse, synchronize, and store the multi-modal data streams. **(B)** Data flow from patients to researchers and clinicians. The data infrastructure pipeline is integrated into research workflows, such that researchers have easy access to patient data but are removed from the process of managing the data transition and ingestion. **(C)** Comparison of local versus cloud-based data management.

The technology’s architecture is organized as a multi-step pipeline (Figure 1A). First, patient data are uploaded—either in batches or in real time—from all devices such as neural implants, wearable monitors, and mobile applications. Data from internet-connected devices such as wearable and mobile devices are uploaded automatically. Data from neural implants can be directly uploaded via HTTPS, or automatically synchronized via third party cloud storage platforms, such as Box, reducing the need for manual file transfer. With each upload, a permanent copy of the original data is securely stored and versioned, and a data catalog is updated to mark its location, ownership, and details. New data are immediately processed upon arrival with a high-availability upload application programming interface (API) that maintains at least 99.9% service uptime. This is achieved with (1) containerized deployment, whereby daily code updates are rolled out with only 10% of containers taken offline at a time, so that the cluster as a whole remains responsive, and (2) service level agreements with Amazon Web Services that guarantees service uptime (AWS Service Level Agreements, 2021).

Then, data pass through an ingestion layer, which parses proprietary or open source data formats and outputs time series signals together with device-related meta data, such as recording configurations (electrode pairs, sampling rate, etc.) and stimulation settings (frequency, amplitude, ramp rates, etc.) (Sellers et al., 2021). Importantly, full integration into both patient and researcher workflows ensures that users need not be involved in the engineering processes that handles data transfer, upload, and parsing (Figure 1B). Patients are primarily tasked with managing devices that collect data, and researchers have access to the resulting time-synchronized datasets, while remaining removed from the engineering details in Figure 1A.

Ingested data are stored in a structured time series database. A distributed Write-Ahead Log (WAL) is used to scale data-write horizontally across compute clusters, acting as a surge-protector so that the system is resilient to spikes of new incoming patient data. The WAL data are dispatched into an indexed time-series data store that aligns the multi-modal streams in the time domain. This layer services real-time random access to any segment of the data, across one or many patients and devices. The same WAL data are concurrently double-dispatched to a replicated data lake, where much larger cross-patient query and analysis can be performed at petabyte scale.

Finally, all data are available through a Python-based API and software development kit (SDK). Because complete datasets are parsed for both time series and meta data components, data are easily queried and accessed for either cloud or local compute (Figure 1C). This reduces the need for research groups to manually inventory and curate data, using variable organization schemes that may hinder reproducibility across teams.

**FIGURE 2:**
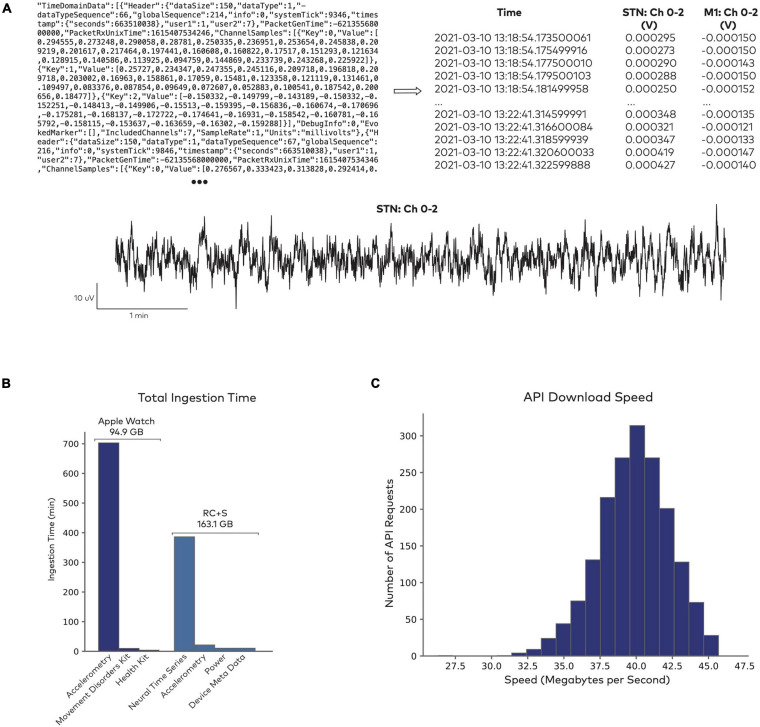
Data access through Rune’s API. **(A)** Sample of raw data from the Summit RC + S system (top left), which gets parsed into a human-readable format and indexed for storage (top right). Both the raw and parsed data formats are accessible for further analysis (bottom). **(B)** Data ingestion performance in sample datasets. Total time for ingesting 10661 Apple Watch datasets, totalling 94.9 GB, and 1983 RC + S datasets, totalling 163.1 GB. Raw data formats are parsed into separate fields, such as accelerometry time series, derived health metrics from the Apple Watch (heart rate, step count, etc.), neural time series, and device meta data. **(C)** Data access through Rune’s API. Distribution of data download speed across 1,800 randomized API requests.

Figure 2A shows an illustrative example of a raw data file from the Medtronic Summit RC + S system and its parsed, human readable format. Given the size and scale of the multimodal continuous time series collected in neuromodulation research, data ingestion represents a large computational effort (Figure 2B). Industry-scale databases offer an efficient, safe, and standardized approach to handling these large datasets. Analysis-ready data are accessible reliably through an API (Figure 2C). To test API performance, we accessed data using 1,800 randomized API requests with a 100% success rate. The mean data request size was 290.3 ± 56.0 MB, with an execution time of 7.3 ± 1.2 s. Combined across all queries, a total of 522.6 GB of data were downloaded in 3.6 h. These performance tests capture a baseline level representative of the platform at its current state. As the platform is updated over time to leverage new designs and tools, performance is expected to continually improve.

The entire system architecture—from ingestion to data access through the API—facilitates data sharing and analysis reproducibility within and between research teams. First, all datasets are treated immutably and versioned, from the raw file through parsing, pre-processing, and post-processing steps. The origin of each initial and intermediate dataset is recorded, including the algorithmic code applied between each input/output layer. This ensures that all versions of accessed data can be traced. Second, data access through an API enables scientific collaborators to share analysis code that is not dependent on local machine file/directory configurations or individual data parsing algorithms.

### Development Process

The Rune Labs platform is developed using industry-level data pipeline tools with leading standards for code documentation, maintenance, and security audits, including secure handling of sensitive patient health information. Amazon Web Services’ set of HIPAA-eligible services guarantee both reliability and compliance. Data at rest are encrypted universally, and all connections inside and out of the network perimeter are secured by Transport Layer Security. All personally identifiable information is isolated to a single part of the platform topology and secured, allowing the vast majority of the internal services to act entirely on de-identified data.

All development is structured through a process of collaborative design, architectural review, automatically enforced code test coverage and quality standards, and explicit security and risk assessment checkpoints. The Standard Development Lifecycle is designed around the FDA-mandated format of validation and verification. Risk matrices are created for all new features, outlining possible failure modes and security attacks, mapped to the corresponding standard FDA severity and probability indices, implemented mitigations, and finally the respective validation and verification over those mitigations. In order to maintain stability as the platform continuously evolves to follow the research community, new functionality is wrapped in conditional execution containers so it can be vetted thoroughly against subsets of patient data before being enabled universally. This latter pattern enables safe and rapid integration of new devices and data types. It therefore ensures a safe, scalable, and efficient solution for data access, aggregation, and sharing.

## Shared Data Platforms and Neuromodulation Therapy Development

Traditionally, neuromodulation researchers have created in-house systems, applications, libraries, and toolkits to manage data generated during clinical trials. However, effectively managing the increasingly large and complex datasets requires a significant investment in software engineering. Furthermore, individual research laboratories may not be equipped for maintenance, compliance, and lifecycle management of data software that has been custom-developed for single trials or projects. Thus the end of a project—or even the departure of a key researcher—can pose a major hurdle for long-term utilization of valuable datasets.

An alternative is for researchers to leverage data platforms that are shared in common with other research groups and clinical teams. These “out-of-the-box” systems have the advantage of long-term stability, compliance, and scalability for patients, clinicians, and researchers. Embracing collaborative data platform ecosystems can save time and eliminate redundancy, accelerating the translation of technologies from laboratory to clinic (Borton et al., 2020). The use of common data platforms may also facilitate open-sourcing de-identified datasets, enabling researchers to combine data from different patients, projects, and research centers.

Existing data sharing options, including both data archives and databases, are not currently optimized for chronic neuromodulation datasets. Data archives serve as a repository for sharing data files, which can include both variable data formats (DABI, 2021) or common data structures (Dandi Archive, 2021). However, unlike databases, archives do not enable efficient data query for the large-scale data that are produced with chronic recordings. Similarly, existing databases in the neurology space were developed to support data in acute or cross-sectional study designs, and they primarily service different data modalities, such as neuroimaging (D’Haese et al., 2015). Chronic multi-modal time series data are not optimally served by existing data archive and database options, though a specific data solution is necessary for managing these growing datasets.

When deciding whether to use a shared data platform to support a project or clinical trial, researchers will have to weigh several factors. Custom, internally developed software may be suitable for small feasibility studies. However, a common data platform offers several advantages. First, a data platform utilizes validated data pipelines for the neuromodulation device, wearable device, or other data source. Multi-modal data sources can be difficult to synchronize and ingest, and validated data pipelines can reduce errors. Second, a common data platform enables the synthesis of datasets across trials, centers, or patient cohorts. Finally, a common data platform facilitates collaborative analysis. Accessing data from the cloud with a documented API/SDK facilitates easy code sharing and ensures that all collaborators are working from the same datasets.

## Conclusion

The future of DBS therapies is shifting toward personalizable, precision medicine. Researchers and clinicians are generating growing datasets that are increasingly difficult to manage and analyze. Here, we described an example of a data infrastructure platform that unblocks access and utilization of complex, multi-modal datasets for researchers and clinicians to develop next-generation neuromodulation therapies. As neuromodulation researchers adopt these types of data platforms for supporting development of new therapies, we can expect larger trials across multiple centers, more reproducibility of key analytical results and programming strategies, and faster discovery of new biomarkers and therapeutic targets.

## Author Contributions

WC wrote the manuscript with support from LK, MK, and BP. RG edited the manuscript. MK designed the data platform. PS assisted with analysis. BP conceived the original idea. All authors contributed to the article and approved the submitted version.

## Conflict of Interest

WC, LK, MK, PS, and BP were employed by Rune Labs. RG was a consultant for Rune Labs.

## Publisher’s Note

All claims expressed in this article are solely those of the authors and do not necessarily represent those of their affiliated organizations, or those of the publisher, the editors and the reviewers. Any product that may be evaluated in this article, or claim that may be made by its manufacturer, is not guaranteed or endorsed by the publisher.
